# A Unique Case of Progressive Hemi-facial Atrophy Successfully Treated with Methotrexate

**DOI:** 10.51894/001c.5783

**Published:** 2017-02-02

**Authors:** Nichelle Arnold, Shahjahan Shareef, Lynn Sikorski

**Affiliations:** 1 Beaumont Health Farmington Hills Dermatology Residency Program, PGY3 Michigan State University; 2 Traditional Rotating Intern Broward Health Medical Center; 3 Beaumont Farmington Hills Dermatology Residency Program, Attending Physician Michigan State University

**Keywords:** parry-romberg syndrome, progressive hemi-facial atrophy, morphea

## Abstract

The effects of many dermatologic syndromes are not exclusive to the skin. Disorders commonly involve a complex interplay between multiple organ systems, thus not relying solely on the dermatologist for proper work up, diagnosis, and treatment. Morphea is one such rare disease which involves progressive loss or atrophy of subcutaneous tissue, muscle, and bone with a relatively mysterious etiology. The initial lesion of morphea can be subtle and appear as a pink to red plaque without any additional symptomatology. A biopsy at this early stage is non-specific and will only show the presence of a T cell infiltrate, vascular swelling, and edema. This active or progressive stage will continue for years before “burning out,” or halting progression, although still affecting underlying tissues. Many times, the sclerosis becomes severe enough to cause deformity and secondary systemic symptoms. Five general subtypes of morphea exist, including: plaque-type, linear, deep, guttate, and nodular. In this paper, the authors report a case report of a rare subtype of linear morphea called Parry Romberg syndrome, also known as progressive hemi-facial atrophy (PHA). PHA usually involves at least one branch of the trigeminal nerve unilaterally. The authors will emphasize the importance of a multidisciplinary approach to diagnose and treat this disorder while also considering the multiple theories surrounding its pathophysiology.

## INTRODUCTION

Parry-Romberg syndrome, or progressive hemifacial atrophy (PHA) is a sporadic, acquired neurocutaneous disorder characterized by progressive hemiatrophy of the skin, soft tissue, muscles, cartilage, and underlying bony structure.^1–3^ The disorder was first described by Parry in 1825 and Romberg in 1846.^4^ The onset of this syndrome typically develops in the first and second decades of life, and more commonly occurs in women.^2,4,5^

Clinically, the disease presents as atrophy of the skin, fat, and osteocartilagenous structures of the lower face. The atrophy typically follows the distribution of the trigeminal nerve. As the disease progresses, hyperpigmentation or depigmentation accompanies the overlying shiny, dry, atrophic skin, and induration ensues. The skin appears adherent or bound down to the underlying structures without the pliable subcutaneous tissue. The lack of underlying tissue generally creates a striking skeleton-like appearance to the affected side of the face. The overlying skin is also commonly either hypopigmented or hyperpigmented, furthering the patient’s cosmetic concerns. The amount of cutaneous deformation is dependent on duration and aggressiveness of the progressive phase.^2–5^

Cicatricial (i.e., sub-epithelial scarring) alopecia of the affected hair-bearing areas is common.^2–5^ PHA more commonly manifests on the left side of face with unclear causality.^1^ Early in the disease process, progression can be halted by topical steroids and/or systemic immune modulators.^2^ Unfortunately, patients commonly present after the disease has “burnt out” or halted in its progression to become more atrophic. Significant atrophy can affect the tongue, gingiva, teeth, and bone causing the face to pull toward the defect. Ipsilateral arm, trunk, and leg may also be involved.

Depending on the extent of the disease, dermatology, neurology, plastic surgery, ophthalmology and oral maxillofacial specialists may need to be involved in developing a plan of care. Through presentation of this unique case report, the authors’ objective is to demonstrate how a multispecialty approach is often necessary to optimally treat PHA. This paper will also include discussion of the etiology, pathophysiology and various treatment options aimed at slowing the progression of the disease and addressing associated cosmetic concerns.

### Case Description

**Figure 1:**
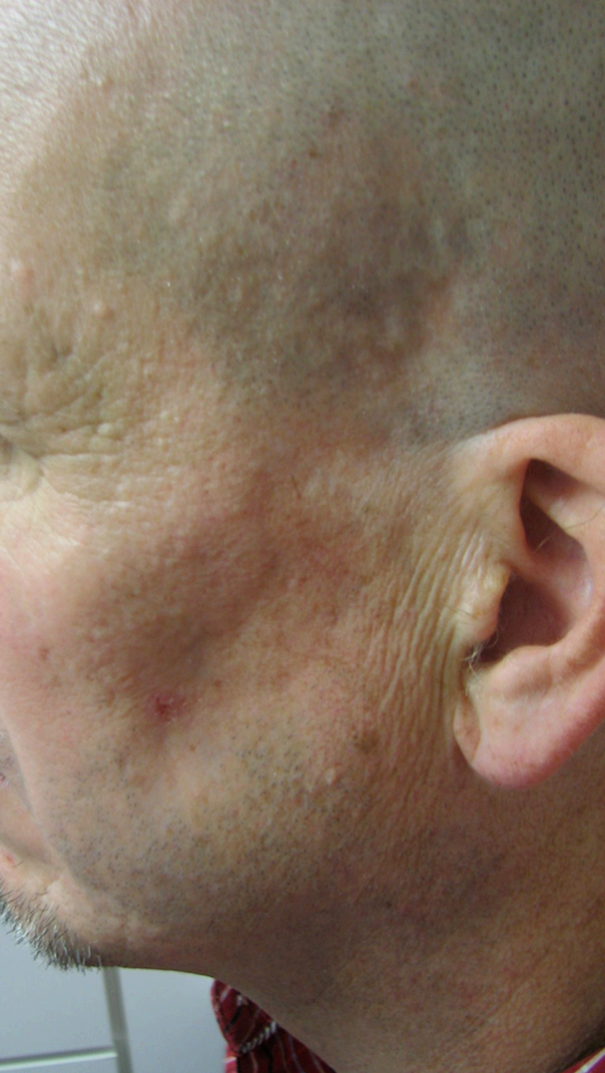
Clinical image demonstrating the atrophic, and hypo pigmented lower cheek/jaw

This patient was a Caucasian male in his 40s who presented to the dermatologist office in 2015 with a chief complaint of hypopigmentation, and atrophy of the skin overlying his left temple and lower jawline (see Figure 1). During the prior two years, the patient reported a gradual loss of subcutaneous tissue, growth of the hypopigmented area, discomfort with mastication, and intermittent “muscle spasms” in his left cheek.

In 2006, he had been initially examined for a patch of hypopigmentation on his left temple. A biopsy was performed, which was consistent with Lichen Sclerosis et Atrophicus or morphea. A thorough examination revealed no other skin or systemic manifestations at that time. Treatment was directed toward the hypopigmentation area with close monitoring for disease progression. The patient’s hypopigmentation improved with a topical steroid and pimecrolimus, and he was subsequently lost to follow-up. Approximately nine years later, the aforementioned skin changes and increasing neurologic symptoms prompted him to seek re-evaluation.

Pertinent review of systems was also positive for joint pain affecting the small joints of his hands. Family history included a mother with osteoarthritis and a father with psoriasis.

Physical exam revealed an approximately 5cm by 3.5cm hypo-pigmented, firm, tightly adherent, or bound down plaque extending from the left temple, to the angle of the mandible and medially to the mid cheek. Atrophy of temporalis and adjacent muscles of facial expression was present (see Figure 1). No fasciculations were observed and sensation was intact. Neurologic exam was otherwise negative and tongue was midline without deficit. Laboratory testing was negative, including markers for autoimmune disease. MRI of the brain with and without contrast revealed no gross mass, white matter changes, or adenopathy.

**Figure 2:**
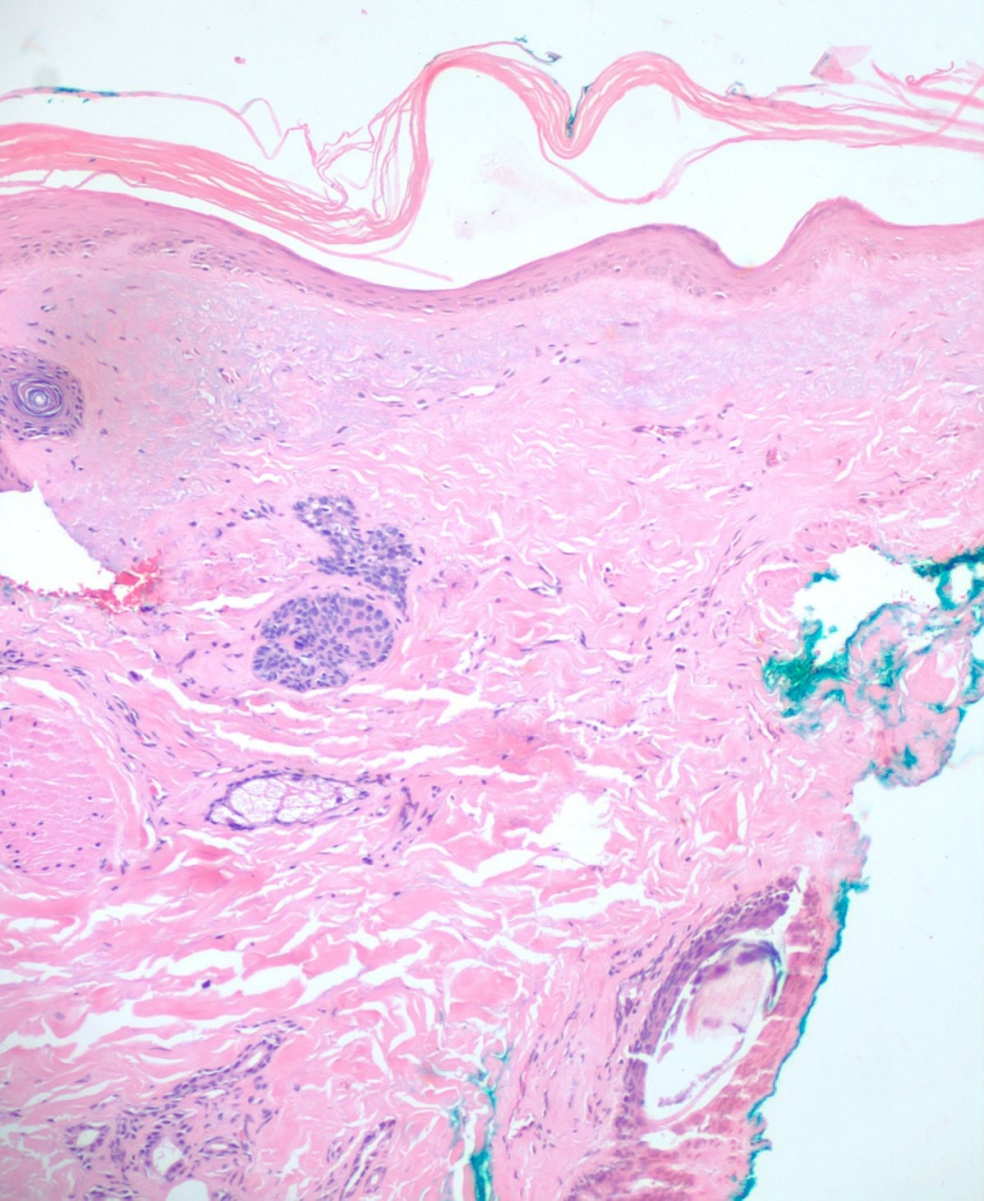
Hematoxalin and eosin Histology image showing atrophy of the epidermis, dermis, and thickening of the collagen with little inflammation

A 4mm. punch biopsy of the atrophic area on the left cheek was completed. Histology was consistent with late stage morphea, characterized by deep dermal sclerosis with thick, hyalinized collagen bundles (see Figure 2). Based on the clinical course and histologic findings, the patient was diagnosed with Parry Rhomberg syndrome, also known as PHA. The patient was referred to a rheumatologist and neurologist for further assessment of associated joint paint and muscle spasms of the jaw. A diagnosis of seronegative rheumatoid arthritis led to the addition of Celebrex for his joint pain. An MRI and EMG were recommended to rule out underlying musculoskeletal involvement. The patient elected to undergo systemic treatment with methotrexate, secondary to rapid advancement of the morphea and musculoskeletal symptoms. After one month of treatment, the hypopigmentation, jaw stiffness, and muscle spasms were subjectively improved.

## DISCUSSION

Although the underlying etiology and pathogenesis of PHA is not well known, it has been hypothesized to be related to trauma, genetics, infection, vascular malformations, endocrine disturbances, increase in cervical sympathetic nerve activity, or auto-immune disorders.^4–6^ Studies report that 24-34% of PHA patients had a history of trauma including: accidental trauma, operative trauma (thyroidectomy and dental extraction), and obstetric trauma (vacuum maneuvers or forceps).^2–4^ Some experts also believe that PHA may be inherited in an autosomal dominant manner with incomplete penetrance.^2,5^

Viruses or bacteria may also play a role in the pathogenesis of some PHA cases.^2^ Herpes zoster, Lyme disease, syphilis, rubella, tuberculosis, otitis media, and diphtheria have all been implicated in the development of PHA. A disorder in the neural crest migration, from which cranial vessels take origin or a chronic cell mediated vascular injury, may also comprise the source of the dysfunction. Abnormal thyroid activity is the most common endocrine disturbance associated with PHA.^2–5^ Thyroid dysfunction may have a negative effect on adipose tissue, leading to atrophy.^2–5,7^

Sympathetic hyperactivity caused by superior cervical ganglion inflammation and irritation has also been hypothesized as a potential pathogenic mechanism leading to development of PHA.^8^ Frequent associations with autoimmune conditions and the detection of autoimmune markers in the blood and cerebrospinal fluid, support the hypothesis that PHA can be triggered by an autoimmune disorder.^2–4^ Pregnancy, stress, and surgery have also been reported to accelerate the disease.^9^ The patient described in this case report denied having sustained any previous trauma to the area, and could not recall if he had developed an infection at the time of initial presentation. He denied having any family history of autoimmune conditions, and his labs (ANA, SCL70, anti-dsDNA, TSH) were negative excluding an underlying autoimmune condition. Therefore, the etiology in this patient’s case is most likely a combination of stress and genetic predisposition.

Systemic manifestations of the disease can include neurologic, psychological ophthalmologic, rheumatologic, cardiologic, endocrine, and maxillofacial abnormalities. Seizures and headaches are the most common neurologic manifestations.^5^ Neurological referral and MRI evaluation are therefore imperative. Ipsilateral hyper-intense white matter lesions is a common MRI finding.^2–5^ Secondary trigeminal neuralgia, dysarthria and or aphasia can often be noted due to destruction of bony structures further impinging on local vasculature.^4,8,10^ Recurrent migraines, cerebral atrophy, hemiplegia, and cranial aneurysms can also occur.^2,3^ Often overlooked, about 46% of patients with PHA experience psychosis and or depression.^1,3^ Therefore, it is important to screen office patients and refer them to appropriate mental health professionals whenever possible. When the authors discussed the diagnosis with this particular patient, he denied having had any feelings of depression or anxiety. It is still important to follow PHA patients closely through treatment and have an open conversation with them during each office visit encounter.

Enopthalomos, uveitis, glaucoma, oculomotor nerve palsies, eyelid atrophy, heterochromia, restrictive strabismus and even blindness are a few of the many ophthalmologic findings sometimes associated with PHA.^1–5,11^ Our patient had recently visited the ophthalmologist and denied any vision changes. Ocular findings are also more common with a V1 trigeminal distribution as the disease can affect the underlying orbital tissues.

A positive anti-nuclear antibody (ANA) is the most common rheumatologic lab abnormality, seen in about 25-52% of PHA cases. Inflammatory bowel disease (5%), rheumatoid arthritis (4%), ankylosing spondylitis (2%), and systemic lupus erythematosus (2%) are the most common autoimmune disorders associated with PHA.^4^ Any suspected clinical indications of hypertrophic cardiomyopathy and/or congenital heart disease should trigger a cardiology consult.^2,12^

Oral manifestations such as angular cheilitis, gingivitis, and absent parotid glands can be appreciated in PHA. Unilateral tongue atrophy, jaw hypoplasia, and a palate deficiency can further result in communication disorders and dysphonia. Tooth and mandibular involvement should be assessed by a general dentist or orthodontist.^3,13^

Once a clinical diagnosis of PHA has been made, a multidisciplinary approach helps ensure that systemic manifestations are evaluated and treated appropriately. The diagnosis of PHA is primarily clinical. Still, biopsies are commonly performed for further diagnostic confirmation. Histopathologic analysis can reveal atrophy of the epidermis, dermis, and adnexal structures, and hypertrophy of collagenous fibers in the superficial dermis is characteristic (see Figure 2).^2,4,5^ As already stated, an MRI, ophthalmologic exam, and rheumatologic screening serology should be performed upon diagnosis. Photographical records from the time of diagnosis are recommended to follow disease progression and duration.^4^ Ultrasound tests can also be used to monitor disease activity and progression by measuring dermal thickness and echogenicity of affected areas. Increase in dermal blood flow can be seen on color doppler as a sign of active disease.^4,14,15^

Treatment of PHA is challenging and aimed at impeding progression of the disease as there are no curative treatments. Initial treatment of lesions in the inflammatory or active stage can be accomplished using high potency topical corticosteroids alternating with a calcineurin inhibitor, which is a non-steroidal inhibitor of T-cells. Topical treatments may take months to show an effect, so close follow-up is recommended with transition to systemic therapy if the disease continues to progress. Standard treatment of late-stage disease is methotrexate with dose ranges from 0.3-1mg/kg/week with a maximum dose of 25 mg weekly either by oral or injection routes. Prednisone (1mg/kg/day) can be added to this regimen for two months with a taper in the third month, due to methotrexate’s delayed effect on inflammation and fibrosis.^4,5,16^

Once disease progression has halted or “burned out,” aesthetic augmentation to restore facial symmetry and volume may be beneficial. Injectable fillers and fat grafting can be used to add volume to the face. If bone is involved, facial plastic surgeons as well as oral maxillofacial specialists work to restore the jaw line. Treatment will involve a multispecialty approach with multiple procedures to correct the appearance, function, and also minimize psychosocial effects. This unique case of a male with PHA highlights how a seemingly specific dermatologic disease truly involves a multidisciplinary approach to care, treatment, and follow-up.

### Conflict of Interest

The authors have no financial or other conflicts of interest to disclose.

## References

[ref-1991] Aydin H., Yologlu Z., Sargin H., Metin M.R. (2015). Parry-Romberg syndrome. Physical, clinical, and imaging features. Neurosci (Riyadh).

[ref-1992] El-Kehdy J., Abbas O., Rubeiz N. (2012). A review of Parry-Romberg syndrome. J Am Acad Dermatol.

[ref-1993] Sommer A., Gambichler T., Bacharach-Buhles M., Rothenburg T., Altmeyer P., Kreuter A. (2006). Clinical and serological characteristics of progressive facial hemiatrophy: A case series of 12 patients. J Am Acad Dermatol.

[ref-1994] Tolkachjov S.N., Patel N.G., Tollefson M.M. (2015). Progressive hemifacial atrophy: A review. Orphanet J Rare Dis.

[ref-1995] Tollefson M.M., Witman P.M. (2007). En coup de sabre morphea and Parry-Romberg syndrome: a retrospective review of 54 patients. J Am Acad Dermatol.

[ref-1996] Trisal D., Kumar N., Dembla G., Sundriyal D. (2014). Parry-Romberg syndrome: uncommon but interesting. BMJ Case Rep.

[ref-1997] Ruhin B., Bennaceur S., Verecke F., Louafi S., Seddiki B., Ferri J. (2000). Progressive hemifacial atrophy in the young patient: Physiopathologic hypotheses, diagnosis and therapy. Rev Stomatol Chir Maxillofac.

[ref-1998] Cory R.C., Clayman D.A., Faillace W.J., McKee S.W., Gama C.H. (1997). Clinical and radiologic findings in progressive facial hemiatrophy (Parry-Romberg syndrome. AJNR Am J Neuroradiol.

[ref-1999] Stone J. (2003). Parry-Romberg syndrome: A global survey of 205 patients using the Internet. Neurology.

[ref-2000] Dalla Costa G., Colombo B., Dalla Libera D., Martinelli V., Comi G. (2013). Parry Romberg syndrome associated with chronic facial pain. J Clin Neurosci.

[ref-2001] Miller M.T., Sloane H., Goldberg M.F., Grisolano J., Frenkel M., Mafee M.F. (1987). Progressive hemifacial atrophy (Parry-Romberg disease). J Pediatr Ophthalmol Strabismus.

[ref-2002] Bucher F., Fricke J., Neugebauer A., Cursiefen C., Heindl L.M. (2016). Ophthalmological manifestations of Parry-Romberg syndrome. Survey of ophthalmology.

[ref-2003] Al-Aizari N.A., Azzeghaiby S.N., Al-Shamiri H.M., Darwish S. (2015). Tarakji B: Oral manifestations of Parry-Romberg syndrome: A review of literature. Avicenna J Med.

[ref-2004] Weibel L., Howell K.J., Visentin M.T., Rudiger A., Denton C.P., Zulian F. (2007). Laser Doppler flowmetry for assessing localized scleroderma in children. Arthritis Rheum.

[ref-2005] Wortsman X., Wortsman J., Sazunic I., Carreno L. (2011). Activity assessment in morphea using color Doppler ultrasound. J Am Acad Dermatol.

[ref-2006] Zulian F., Athreya B.H., Laxer R., Nelson A.M., Oliveira S.K., Punaro M.G. (2006). Juvenile localized scleroderma: Clinical and epidemiological features in 750 children. An international study. Rheumatol (Oxford).

